# Beyond Vitamin D Deficiency: Maternal Diet, Metabolic Dysfunction, and Vitamin D Signaling in Developmental Programming

**DOI:** 10.3390/metabo16100722

**Published:** 2026-09-28

**Authors:** Rachel Rousseau, Chin May Teoh, Gar Yee Koh

**Affiliations:** School of Family and Consumer Sciences, Texas State University, 601 University Drive, San Marcos, TX 78666, USA

**Keywords:** maternal nutrition, Western diet, developmental programming, vitamin D signaling, vitamin D receptor, metabolic inflammation, tissue-specific regulation

## Abstract

Within the Developmental Origins of Health and Disease (DOHaD) framework, maternal nutrition is an important component of the developmental environment that can shape long-term offspring metabolic and immune health. Western dietary patterns (WD), characterized by high energy density and greater consumption of saturated fats, refined carbohydrates, added sugars, and ultra-processed foods, are associated with metabolic and inflammatory disturbances that may influence fetal development. Experimental models further demonstrate that maternal high-fat and WD exposures can produce persistent metabolic alterations in offspring, supporting a role for maternal dietary composition in developmental programming. Vitamin D (VD) represents a potentially important regulatory system within this environment because VD signaling contributes to immune regulation, cellular differentiation, and metabolic homeostasis through vitamin D receptor (VDR)-mediated transcription. Although circulating 25-hydroxyvitamin D [25(OH)D] is the primary clinical indicator of VD status, effective VD activity also depends on tissue distribution, enzymatic metabolism, and VDR-mediated responsiveness. This review examines evidence linking maternal Western and high-fat dietary exposures with disruption of VD regulation during development, with particular emphasis on potential tissue-specific effects in offspring. Evidence across obesity and high-fat diet models, including maternal exposure models, suggests that metabolic dysfunction may influence several aspects of VD regulation, while maternal VD status has independently been associated with metabolic and developmental outcomes in offspring. However, evidence directly linking maternal WD exposure with altered VD regulation in individual offspring tissues remains limited. Consequently, it remains unclear whether maternal WD exposure produces persistent, tissue-specific alterations in offspring VD regulation and whether these changes occur alongside inflammatory dysregulation. Addressing this gap is particularly important because tissues differ in their expression of VDR and VD-metabolizing enzymes and may therefore respond differently to maternal metabolic stress.

## 1. Introduction

The Developmental Origins of Health and Disease (DOHaD) framework proposes that environmental exposures during critical windows of development, including gestation and early postnatal life, can shape long-term disease susceptibility [1]. This concept is consistent with the “thrifty phenotype” hypothesis, which proposes that the developing fetus adapts to the maternal environment in ways that may influence susceptibility to metabolic disease later in life [2,3]. Experimental and epidemiological evidence demonstrates that adverse early-life conditions can alter developmental trajectories, contributing to long-term changes in metabolic, cardiovascular, and endocrine function [4,5]. Epigenetic processes, including DNA methylation, histone modification, and non-coding RNA regulation, represent one mechanism through which developmental exposures may become biologically embedded by producing persistent changes in gene regulation without altering the underlying DNA sequence [1,6]. Maternal nutrition is particularly relevant within the DOHaD framework because it contributes to the metabolic, inflammatory, and nutrient environment in which fetal development occurs [1]. Disruption of this maternal environment may therefore influence developmental processes with effects extending beyond the prenatal period. Maternal overweight and obesity represent important examples of such altered maternal conditions and have been consistently associated with adverse pregnancy outcomes and increased metabolic risk in offspring [7,8]. Maternal dietary exposure represents a related but distinct component of the developmental environment that can influence maternal metabolic conditions independently of body weight. Although maternal dietary patterns are considered broadly, this review focuses primarily on Western and high-fat dietary exposures because of their relevance to maternal metabolic dysfunction and the proposed convergence with VD regulation during development. As a narrative review, relevant literature was identified primarily through PubMed using Medical Subject Headings (MeSH) and keyword-based searches related to maternal dietary exposure, Western and high-fat diets, vitamin D metabolism and signaling, developmental programming, and offspring outcomes. Titles and abstracts were screened for relevance to the scope of the review. Additional studies were identified through reference lists, related-article searches, and citation tracking of relevant publications. Google Scholar and AI-assisted search tools were also used to identify potentially relevant literature, with retrieved studies subsequently evaluated for relevance to the review topic.

## 2. Maternal Obesity and Offspring Metabolic Programming

Maternal overweight and obesity are associated with metabolic and inflammatory disturbances during pregnancy that may alter the intrauterine environment in which fetal development occurs [9,10]. Compared with lean pregnant women, pregnant women with obesity have been shown to exhibit greater insulin resistance and differences in circulating metabolic and inflammatory mediators, including insulin, leptin, TNF-α, IL-6, and IL-8 [9,10]. Evidence of inflammatory differences also extends to the placenta. Yang et al. reported increased placental *TLR4*, *IL6*, and *IL8* expression in women with obesity, with placental *TLR4* expression positively associated with maternal pre-pregnancy BMI and circulating metabolic and inflammatory markers [10]. Differences in maternal metabolic and inflammatory status, together with altered placental inflammatory signaling, may modify the environment experienced by the developing fetus, providing a potential pathway linking maternal metabolic status with fetal development [10]. Maternal overweight and obesity have also been associated with adverse perinatal outcomes, including preterm birth and excessive fetal growth [11,12]. Gestational weight gain outside recommended ranges has similarly been associated with adverse infant outcomes, including preterm birth, macrosomia, and small-for-gestational-age birth [13,14]. Associations between maternal weight status and offspring health extend beyond the perinatal period. Chang et al. reported that maternal overweight or obesity and excessive gestational weight gain were each associated with greater risk of offspring overweight and obesity at 2 years of age, with the greatest risk observed when both were present [15]. Evidence across childhood and adolescence further demonstrates associations between maternal overweight and obesity and increased offspring risk of overweight and obesity [16,17]. In a large individual participant data meta-analysis including more than 160,000 mother-child pairs, associations between maternal pre-pregnancy BMI and offspring BMI strengthened with increasing offspring age, suggesting that differences associated with maternal weight status may become increasingly apparent across development [17].

Evidence of longer-term associations is not limited to offspring adiposity, with studies also reporting metabolic and inflammatory differences later in life [18,19]. A cross-sectional study of 12-year-old children, for example, reported higher hs-CRP concentrations among those born to mothers with higher BMI, suggesting that maternal weight status may be associated with differences in offspring inflammatory status [19]. Maternal weight status may extend beyond the intrauterine environment to differences in postnatal nutrition; Andersen et al. reported that infants born to mothers with obesity consumed less healthy dietary patterns at 9 months of age, characterized by lower fiber intake, lower fruit and vegetable consumption, and a greater proportion of energy from protein compared with infants born to mothers in the predominantly non-obese comparison cohort [20]. Evidence across the perinatal period, childhood, and adolescence therefore links maternal overweight and obesity with adverse offspring outcomes extending well beyond birth [12,16,17]. However, maternal body weight represents a complex phenotype influenced by multiple dietary, metabolic, behavioral, and environmental factors and does not, by itself, identify the specific exposures responsible for developmental programming. Maternal dietary intake is one modifiable component of this environment, with dietary patterns during pregnancy associated with maternal and offspring health outcomes [1,21]. Examining maternal dietary patterns in addition to maternal body weight may therefore provide greater insight into the environmental exposures and biological pathways contributing to offspring metabolic programming.

## 3. Maternal Dietary Patterns and Offspring Metabolic Programming

Maternal dietary intake represents an important determinant of the intrauterine environment beyond maternal body weight alone. Dietary quality influences maternal metabolic homeostasis, inflammatory status, and nutrient availability throughout pregnancy, thereby shaping the conditions under which fetal development occurs [1,21,22]. Protective, nutrient-dense dietary patterns are generally characterized by greater consumption of fiber, micronutrients, and unsaturated fats, with lower consumption of refined carbohydrates and ultra-processed foods [21,23,24]. Greater adherence to protective dietary patterns during pregnancy has been associated with more appropriate gestational weight gain and lower risks of adverse pregnancy outcomes, including gestational diabetes mellitus (GDM) and preterm birth [21,22,25]. In contrast, Western dietary patterns (WD) are characterized by high energy density and greater consumption of saturated fats, refined carbohydrates, added sugars, and ultra-processed foods [23,24,25]. Human studies associate WD and other energy-dense, nutrient-poor dietary patterns with metabolic disturbances during pregnancy [22,25]. A systematic review of 11 studies found that greater adherence to healthy and traditional dietary patterns characterized by fruits, vegetables, nuts, and dairy was associated with less excessive gestational weight gain, whereas WD and mixed patterns rich in ultra-processed foods were associated with greater excessive gestational weight gain [22]. Similarly, a meta-analysis of 21 prospective cohort studies including 191,589 women found greater GDM risk associated with several foods characteristic of WD patterns, including fast food, animal meat, red meat, and processed red meat [25]. While human studies demonstrate associations between maternal dietary patterns and pregnancy outcomes, preclinical models allow the developmental effects of maternal dietary exposure and their underlying biological pathways to be examined under controlled conditions. The placenta represents one potential pathway linking maternal dietary exposure with fetal development because it regulates the exchange of nutrients and metabolic signals between the maternal and fetal environments. In C57BL/6J mice, placentas from HFD-fed dams exhibited changes in inflammatory and immune-related signaling, including reduced transcript levels of *Traf6*, *Myd88*, *Mapk8*, and *Stat3*, alongside increased expression of immune-cell markers such as *Cd4* and *Cd25* [26]. Maternal HFD has also been associated with placental hypoxia and vascular changes, including increased expression of carbonic anhydrase IX, VEGF, and CD31 and reduced blood vessel maturity [26,27]. In addition, increased placental expression of the glucose transporters GLUT1 (*Slc2a1*) and GLUT3 (*Slc2a3*) and the amino acid transporter SNAT2 (*Slc38a2*) suggests that maternal HFD may influence nutrient transport at the maternal-fetal interface [26]. Altered placental inflammatory signaling, vascular development, and nutrient transport provide potential pathways through which maternal dietary exposure may modify the metabolic and nutrient environment experienced by the developing fetus.

Maternal HFD exposure has also been associated with changes in hepatic metabolic regulation in developing and adult offspring [26,28]. In fetal offspring exposed to maternal HFD, Wallace et al. reported reduced hepatic expression of the gluconeogenic genes *Pc* and *Pepck* and increased expression of the metabolic regulators LXRα (*Nr1h3*) and LXRβ (*Nr1h2*) compared with controls [26]. Evidence of persistent hepatic gene-expression and DNA-methylation differences in adult offspring further indicates that maternal HFD exposure can influence hepatic metabolic regulation across developmental stages [28], these findings indicate that maternal HFD exposure can influence hepatic metabolic regulation across developmental stages. Responses to maternal HFD may also differ by sex, although the affected pathways vary according to the tissue, developmental stage, and postnatal dietary exposure examined. Gohir et al. reported increased NF-κB activation and decreased unfolded protein response signaling in the fetal gut, with effects particularly evident in female fetuses, whereas Keleher et al. found that maternal HFD exacerbated adult obesity-related phenotypes primarily in HFD-fed female offspring [27,28]. In contrast, Chang et al. observed greater postnatal adiposity, impaired glucose tolerance, and adipose inflammatory responses predominantly in male offspring following maternal HFD exposure and a later postnatal HFD challenge [18]. The persistence of molecular and metabolic differences across developmental stages and tissues suggests that maternal dietary exposure can have lasting effects on offspring gene expression and metabolic function. Epigenetic processes provide one potential mechanism underlying such developmental programming. DNA methylation, histone modification, and non-coding RNA mechanisms can influence gene expression without altering the underlying DNA sequence and are sensitive to nutritional and metabolic conditions [6,29]. DNA methylation is particularly important during cellular differentiation and tissue development and depends in part on methyl groups generated through one-carbon metabolism from nutrients such as folate, choline, B vitamins, and methionine [6]. Histone modifications are similarly influenced by metabolites such as acetyl-CoA and NAD^+^, which reflect cellular nutrient and energy status [6,29,30,31]. Maternal nutrient availability and metabolic stress may therefore shape developmental epigenetic processes through multiple nutrient-sensitive pathways.

Preclinical and human studies provide evidence consistent with a relationship between maternal metabolic conditions and offspring epigenetic regulation. In an overfeeding-induced maternal obesity rat model, male offspring exhibited differences in DNA methylation compared with offspring of control dams, including hypomethylation of genes associated with the adipogenic regulators C/EBP-β, *Zfp423*, and PPARγ [32]. In a mouse model, Keleher et al. similarly reported differences in adult offspring hepatic gene expression and DNA methylation following maternal HFD exposure compared with controls [28]. Human studies provide complementary evidence linking maternal metabolic conditions with offspring epigenetic variation. Maternal GDM and dysglycemia have been associated with differential DNA methylation in infant cord blood, including changes in loci and regulatory networks involved in cell signaling and chromatin remodeling [33]. In the UPBEAT cohort, Antoun et al. identified 242 cord-blood CpGs associated with maternal GDM and additional methylation differences associated with continuous measures of maternal glucose; GDM-associated methylation signals were substantially less apparent in the lifestyle-intervention arm than in the standard-care arm [33]. Dietary intervention studies nevertheless provide variable evidence. A maternal low-glycemic-index intervention within the ROLO cohort was associated with subtle differences in neonatal DNA methylation across multiple genes involved in cardiac, immune, and metabolic pathways. However, the initial findings were not replicated in an additional sample from the cohort [30]. In contrast, the LIMIT randomized controlled trial found no significant cord-blood DNA methylation differences associated with either the antenatal diet and lifestyle intervention or maternal early-pregnancy BMI [34]. In non-human primates, maternal WD exposure was associated with differences in placental and fetal hepatic miRNA profiles compared with control-diet exposure, suggesting that dietary programming may involve multiple forms of regulatory change across the maternal-placental-fetal axis [35]. Maternal dietary exposure may contribute to the developmental environment through interconnected metabolic, inflammatory, placental, and epigenetic pathways [26,28,35]. WD patterns are particularly relevant because they combine excess energy and poor dietary quality with metabolic and inflammatory disturbances that may influence nutrient availability and cellular signaling during development [1,24]. Among the nutrient-sensitive pathways potentially affected by this environment, vitamin D signaling is of particular interest because of its roles in metabolic and immune regulation [6]. Understanding normal vitamin D metabolism and signaling therefore provides a basis for examining how maternal WD exposure may influence this pathway during development.

## 4. Vitamin D Metabolism and Signaling

Given the influence of maternal dietary and metabolic conditions on nutrient-sensitive developmental pathways, vitamin D (VD) represents a particularly relevant regulatory system to consider. Although traditionally recognized for its role in calcium homeostasis and skeletal development, VD also regulates gene transcription across multiple tissues, contributing to immune function, cellular proliferation and differentiation, as well as other extra-skeletal processes [36,37]. These biological effects depend on the coordinated synthesis, transport, metabolism, and receptor-mediated action of VD, which collectively determines systemic and tissue-specific VD activity [36,37]. VD is a fat-soluble secosteroid obtained through cutaneous synthesis and dietary intake. Endogenous production begins when ultraviolet B radiation converts 7-dehydrocholesterol in the skin to previtamin D_3_, which subsequently isomerizes to cholecalciferol (vitamin D_3_) [36,38]. Dietary VD is absorbed in the small intestine with dietary lipids, incorporated into chylomicrons, and transported through the lymphatic system before entering the circulation [39]. Following cutaneous synthesis or intestinal absorption, VD undergoes hydroxylation primarily in the liver, where CYP2R1 converts vitamin D_3_ and vitamin D_2_ to 25-hydroxyvitamin D [25(OH)D], the major circulating metabolite and clinical indicator of VD status [36,40]. Most circulating 25(OH)D is bound to vitamin D-binding protein (VDBP), with smaller fractions bound to albumin or present in the unbound state [40]. These circulating pools provide substrate for the subsequent production of biologically active VD. In the kidney, 25(OH)D is converted by CYP27B1 to 1,25-dihydroxyvitamin D [1,25(OH)_2_D], the hormonally active form of VD [36,37]. CYP27B1 is also expressed in extra-renal tissues and cell types, permitting local production of 1,25(OH)_2_D and contributing to tissue-specific regulation of VD activity [36,37].

The biological actions of 1,25(OH)_2_D are mediated primarily through binding to the vitamin D receptor (VDR), a nuclear receptor that regulates transcription of VD-responsive genes [36,41]. VDR and VD-metabolizing enzymes are distributed across numerous tissues and cell types, including the intestine, kidney, pancreatic β-cells, adipose tissue, immune cells, and placenta, supporting systemic and tissue-specific regulation of VD activity [36,41,42,43]. VDR expression, specifically, is comparatively low or less consistently demonstrated in tissues such as adult liver and skeletal muscle [41]. Through VDR-mediated transcription, VD participates in the regulation of mineral homeostasis, immune responses, cellular proliferation, differentiation, and metabolic processes [36,37,41]. Clinical evidence from multiple sclerosis further illustrates the potential relevance of this pathway. In a recent umbrella review, Abbasi et al. evaluated evidence relating serum 25(OH)D concentrations and VD supplementation to multiple sclerosis, together with genetic variation in *VDR* [44]. Common *VDR* polymorphisms, including ApaI (rs7975232), BsmI (rs1544410), TaqI (rs731236), and FokI, have been investigated because variation in *VDR* may influence receptor function and the expression of VD-responsive genes involved in immune and inflammatory regulation. However, associations between individual VDR polymorphisms and multiple sclerosis were not consistent across genetic models, indicating that their contribution is more complex than a direct relationship between a single variant and disease risk [44].

More broadly, the distribution of VDR and VD-metabolizing enzymes across tissues permits local regulation of VD activity according to tissue-specific physiological demands [37,40,41]. VD activity is further controlled through coordinated regulation of activation and degradation. CYP24A1 initiates the catabolism of both 25(OH)D and 1,25(OH)_2_D, thereby contributing to the regulation of systemic and local VD activity [36,45]. In the kidney, CYP27B1 and CYP24A1 are tightly regulated by endocrine signals involved in mineral homeostasis. Parathyroid hormone stimulates renal CYP27B1, whereas fibroblast growth factor 23 suppresses CYP27B1 and promotes CYP24A1. In contrast, extra-renal VD metabolism is regulated more locally and does not necessarily follow the same endocrine regulatory mechanisms, allowing VD activity to respond to tissue-specific physiological signals [43,45]. The balance between activation and degradation therefore contributes to the regulation of active VD availability, while extra-renal expression of these enzymes permits additional tissue-specific control [37,43]. VD signaling depends on more than circulating 25(OH)D concentrations alone [46,47,48]. Effective VD activity requires adequate metabolite availability and distribution, appropriate enzymatic metabolism, and functional VDR-mediated transcription within target tissues [37,40,48]. Disruption at any of these regulatory levels could therefore alter tissue-level VD activity in ways that may not be fully reflected by circulating 25(OH)D concentrations [37,40]. This integrated regulatory framework provides the basis for examining potential points of convergence between maternal dietary and metabolic exposures, VD regulation, and developmental programming.

## 5. Potential Disruption of Vitamin D Signaling in Western Diet

WD patterns create a metabolic environment characterized by excess energy availability, altered lipid metabolism, chronic low-grade inflammation, and oxidative stress, conditions that may interfere with multiple components of VD regulation [24,49,50]. Obesity and HFD exposure have been associated with reduced circulating 25(OH)D concentrations and altered VD metabolism or tissue distribution [51,52,53]. In both experimental and human studies, obesity-associated reductions in circulating 25(OH)D have been observed despite equivalent or greater VD exposure, suggesting that inadequate intake alone does not fully explain lower VD status in obesity [51,52,53,54]. Differences in VD tissue distribution and enzymatic metabolism provide additional potential mechanisms [51,52,53]. Evidence that metabolic conditions associated with WD and HFD exposure can influence VD distribution and metabolism provides a basis for examining whether similar disturbances within the maternal environment may affect VD regulation during development. The evidence considered in this section addresses distinct but complementary aspects of the proposed framework. Maternal WD/HFD studies that directly assess VD-related outcomes in offspring provide the most direct evidence for developmental effects on VD regulation. In contrast, obesity and non-pregnancy HFD models provide mechanistic evidence for how metabolic dysfunction can influence VD availability, distribution, metabolism, or signaling, but do not establish that the same changes occur during maternal dietary exposure or persist in offspring. Maternal vitamin D deficiency (VDD) and VD supplementation studies address a separate question by demonstrating developmental consequences of modifying maternal VD availability rather than the effects of maternal WD/HFD exposure itself.

### 5.1. Altered Vitamin D Availability and Tissue Distribution

One mechanism through which obesity and HFD exposure may reduce circulating VD availability is altered distribution between circulating and tissue compartments. VD is fat soluble, and expansion of adipose tissue may increase its distribution into adipose stores and reduce the proportion remaining in circulation [52]. In C57BL/6 mice fed either a control diet (10% kcal from fat) or HFD (45% kcal from fat) with varying levels of VD supplementation for 13 weeks, HFD-fed animals exhibited lower circulating 25(OH)D concentrations under VD-supplemented conditions despite greater VD intake [52]. Under the same supplemented conditions, HFD-fed animals also exhibited greater VD_3_ accumulation in the liver and adipose tissue, supporting altered tissue distribution as a potential contributor to lower circulating VD availability [52]. Although this model did not examine pregnancy or developmental exposure, it provides mechanistic evidence that reductions in circulating VD under conditions of HFD-induced obesity may reflect altered tissue distribution and storage rather than inadequate intake alone. Human evidence is consistent with the possibility that obesity-associated reductions in circulating VD are not explained solely by dietary intake. Karlsson et al. reported lower first-trimester VD status among women with obesity than among women with normal weight despite greater reported dietary VD intake [54]. Josefson et al. further reported lower cord-blood 25(OH)D concentrations among neonates born to mothers with obesity than among those born to mothers with normal weight, despite similar maternal 25(OH)D concentrations late in pregnancy [55]. Maternal and neonatal 25(OH)D concentrations are positively correlated, consistent with maternal circulating 25(OH)D serving as an important determinant of fetal VD availability [56]. The findings suggest that obesity-associated differences in VD availability may involve both maternal circulating status and fetal VD exposure [54,55,56].

### 5.2. Impaired Vitamin D Metabolism and Bioactivation

Obesity and HFD exposure may also interfere with the enzymatic pathways responsible for VD metabolism and activation [51]. In C57BL/6N mice with HFD-induced obesity, reduced circulating 25(OH)D concentrations were accompanied by marked suppression of hepatic *Cyp2r1* mRNA expression and CYP2R1 protein abundance, with protein abundance reduced by up to 69% [51]. CYP2R1 is the major hepatic VD 25-hydroxylase; its suppression therefore provides a potential mechanism through which HFD-induced obesity may contribute to reduced circulating 25(OH)D [51]. *Cyp2r1* mRNA downregulation was also reported in extrahepatic tissues, including the kidney and brown adipose tissue, suggesting that HFD-induced metabolic stress may influence VD metabolism across multiple tissue compartments [51]. Consistent with metabolic regulation of this pathway, subcutaneous adipose *CYP2R1* expression increased approximately 1.5-fold following substantial weight loss after gastric bypass surgery in four women with obesity, further suggesting that *CYP2R1* expression is responsive to metabolic state [51].

Evidence from pregnancy models suggests that maternal metabolic dysfunction may also affect VD regulation at the maternal-fetal interface. The placenta expresses key components of the VD metabolic and signaling pathway, including CYP27B1, CYP24A1, and VDR, permitting local regulation of VD activity [53,56]. In a non-human primate model, placentas from diet-induced obese baboons exhibited decreased expression of *LRP2* (megalin), *CYP27B1*, and *VDR*, a reduced *CYP27B1*:*CYP24A1* ratio, and lower nuclear and chromatin-bound VDR protein abundance compared with placentas from lean controls [53]. Reduced *LRP2*, *CYP27B1*, and *VDR* expression implicates multiple components of placental VD regulation, including uptake of VDBP-bound 25(OH)D, local conversion of 25(OH)D to 1,25(OH)_2_D, and receptor-mediated signaling [53]. Human studies further demonstrate relationships between placental VD metabolism and the maternal environment. Placental CYP27B1 protein abundance has been positively associated with maternal 25(OH)D concentrations at both mid-gestation and delivery [56]. Inflammatory status was also related to placental VD metabolism, with neonatal IL-6 concentrations negatively associated with placental *CYP24A1* mRNA expression [56]. Although these observational associations do not establish that maternal VD or inflammatory status directly regulates placental VD metabolism, they indicate that placental VD regulatory pathways vary in relation to VD availability and inflammatory status. Maternal metabolic conditions may therefore influence fetal VD exposure through circulating substrate availability and local regulation of VD uptake, activation, and signaling at the placenta.

### 5.3. Inflammation- and Oxidative Stress–Associated Alterations in Vitamin D Responsiveness

A third potential level of disruption occurs downstream of VD availability and enzymatic activation, at the level of cellular responsiveness. Obesity and WD exposure are associated with chronic low-grade inflammation and oxidative stress, particularly within expanded adipose tissue [49,50,57]. In male C57BL/6J mice, consumption of a 60% fat diet for 16 weeks increased adipose NF-κB activation, macrophage recruitment and crown-like structure formation, and expression of pro-inflammatory mediators, including TNF-α and IL-6, compared with lean controls [57]. These findings demonstrate an inflammatory adipose environment within which cellular VD signaling must function. VD supplementation in HFD-fed mice reduced NF-κB activation, expression of NLRP3 inflammasome-related genes, pro-inflammatory cytokine expression, and macrophage recruitment in adipose tissue [57]. In cultured human adipocytes, 1,25(OH)_2_D_3_ suppressed IL-6 and leptin expression, along with NF-κB and ERK1/2 phosphorylation; these inhibitory effects were blocked by VDR knockdown, supporting VDR-dependent regulation of inflammatory signaling [58,59]. The biological actions of VD depend in part on VDR-mediated transcription; alterations in receptor function or downstream transcriptional processes could therefore modify cellular responsiveness to VD [58,59].

Oxidative stress represents another potential contributor because intracellular redox conditions can influence nuclear-receptor function and transcriptional regulation. Experimental evidence indicates that oxidative and nitrosative stress can inhibit VDR–RXR DNA binding and transcriptional activity, providing a potential mechanism through which metabolic oxidative stress could impair VDR-mediated responsiveness [60,61]. Thus, inflammatory and oxidative conditions may represent an additional level at which metabolic stress modifies VD-responsive pathways downstream of circulating VD availability and enzymatic activation. WD-associated metabolic dysfunction may interfere with VD regulation at multiple biological levels. Altered tissue distribution may reduce circulating VD availability, dysregulation of VD-metabolizing enzymes may impair production and local activation of VD metabolites, and inflammatory or oxidative conditions may modify cellular VD responsiveness [51,52,53,57,58,59]. These mechanisms are not mutually exclusive and may occur simultaneously within the maternal-placental-fetal environment, providing a potential framework through which maternal WD-associated metabolic stress could influence VD regulation during development (Figure 1). Consequently, circulating 25(OH)D concentrations alone may not fully characterize the effects of maternal metabolic stress on VD biology during development [40,51,52,53]. The developmental significance of these disruptions depends, however, on whether alterations in the maternal VD environment are capable of producing persistent changes in offspring metabolic regulation.

#### 5.3.1. Developmental Consequences of Altered Maternal Vitamin D Signaling

Evidence that maternal WD-associated metabolic dysfunction may alter VD availability, metabolism, and cellular responsiveness raises an important question regarding the developmental consequences of disrupted VD signaling. Maternal VD status is closely related to fetal VD exposure, and lower maternal 25(OH)D concentrations during pregnancy have been associated with differences in offspring adiposity [56,62,63]. In the GUSTO cohort, lower maternal mid-gestation 25(OH)D concentrations were associated with greater neonatal abdominal subcutaneous adiposity, including both superficial and deep subcutaneous adipose tissue [63]. Longitudinal studies have similarly reported associations between lower maternal 25(OH)D concentrations and greater offspring BMI and waist circumference during childhood [62]. Although these observational findings cannot establish causality, they support a relationship between the maternal VD environment and offspring adiposity. Experimental models provide stronger evidence that disruption of maternal VD availability during development can influence offspring adipose biology. Importantly, VDD models do not replicate WD-induced VD dysregulation but instead establish the developmental consequences of disrupting maternal VD availability. In male mouse offspring, Belenchia et al. found that prenatal VDD produced a greater visceral-to-subcutaneous adipose tissue ratio and a 3.7-fold increase in perigonadal adipose *Pparg* expression compared with offspring from VD-sufficient dams [64]. *Pparg* encodes PPARγ, a transcriptional regulator of adipocyte differentiation and lipid storage. Prenatal VDD also increased susceptibility to adipocyte hypertrophy following postweaning HFD exposure [64]. In a separate study, Belenchia et al. reported increased perigonadal adipose *Pparg* and *Vdr* expression in lean male offspring despite no significant differences in body weight, adipose-pad weight, or adipocyte size [65]. Although the offspring were VD sufficient at the time of assessment, circulating concentrations of the adipokine resistin and the inflammatory cytokine IL-2 were also higher following maternal VDD exposure [65].

In rats, maternal VDD initiated before conception and maintained throughout gestation did not alter offspring birth weight compared with controls; however, offspring exposed to maternal VDD exhibited greater body weight from approximately 10 weeks of age, accompanied by increased adipose tissue volume, differences in blood lipids, and higher peak blood glucose later in development [66]. Adipose-derived precursor cells from offspring exposed to maternal VDD also exhibited increased proliferation and lipid accumulation, together with widespread DNA methylation and transcriptional differences in genes involved in metabolic regulation [66]. The delayed emergence of these phenotypes despite similar birth weight is consistent with the DOHaD framework, in which developmental exposures may produce latent alterations that become apparent later in life. The persistence of metabolic alterations after prenatal VD disruption raises the possibility that VD signaling interacts with epigenetic processes involved in developmental programming. Supporting evidence outside pregnancy indicates that components of the VD pathway can be epigenetically regulated. Meyer et al. demonstrated that DNA methylation of the *VDR* gene was associated with variation in VDR expression and downstream TLR2/1–VDR signaling, including transcriptional activation of the VDR target gene *CAMP* [67]. Additional evidence linking VD status with epigenetic variation comes from an integrated genomic and epigenomic analysis of African American adolescents with severe VDD, which identified methylation differences at VD-related loci, including *DHCR7*, *CYP2R1*, and *CYP24A1* [68]. Although these studies do not address maternal exposure or developmental programming, they provide supporting evidence for interactions between VD biology and epigenetic regulation. More directly relevant to development, the MAVIDOS randomized controlled trial found that maternal cholecalciferol supplementation from 14 weeks of gestation to delivery was associated with lower DNA methylation at specific CpG sites near the *RXRA* promoter in umbilical cord tissue [69]. RXRα heterodimerizes with VDR during transcriptional regulation of VD-responsive genes, providing a potential link between maternal VD exposure and epigenetic regulation of the VD signaling pathway [69]. Although direct evidence linking maternal WD, altered VD signaling, and offspring epigenetic programming remains limited, these findings support potential interactions between VD signaling and epigenetic regulation during development. Collectively, human and experimental evidence suggests that disruption of the maternal VD environment may have consequences extending beyond circulating VD concentrations during pregnancy. Prenatal VDD has been associated with later differences in offspring adiposity, glucose and lipid metabolism, inflammatory regulation, and gene expression, with some phenotypes becoming apparent only later in development [64,65,66]. These findings support the possibility that altered VD signaling during critical developmental windows contributes to long-term metabolic programming. The persistence of these effects after prenatal exposure has ended also raises the question of whether restoration of VD availability alone is sufficient to reverse developmental changes once they have been established.

#### 5.3.2. Vitamin D Supplementation and Developmental Outcomes

Given the associations between maternal VD status and offspring development, VD supplementation during pregnancy has been investigated as a strategy to improve maternal and neonatal VD status and potentially reduce adverse developmental outcomes. Recent synthesis of systematic reviews and meta-analyses further indicates that maternal VD status and supplementation have been examined across a broad range of maternal and offspring health outcomes, although the strength of evidence varies by outcome [70]. Randomized controlled trials consistently demonstrate that prenatal VD supplementation increases maternal and cord-blood 25(OH)D concentrations [71,72]. In the double-blind trial by Hollis et al., higher-dose VD supplementation produced progressively higher maternal 25(OH)D concentrations, confirming that maternal biochemical VD status responds to supplementation [71]. However, improvements in biochemical VD status have not consistently translated into corresponding improvements in pregnancy or neonatal outcomes. Systematic review evidence indicates variable effects of prenatal VD supplementation on clinical outcomes, including birth weight, preterm birth, gestational diabetes, and preeclampsia, with interpretation limited by differences in trial design, outcome ascertainment, supplementation regimen, and study quality [72,73]. Experimental evidence further suggests that developmental responses to VD intervention may depend on the form and dose administered. In Brown Norway rats exhibiting pregnancy-specific VD deficiency associated with dysregulated VD metabolism, increasing dietary cholecalciferol increased circulating 25(OH)D but did not improve litter size or fetal and placental weights. In contrast, low-dose treatment with the active metabolite 1,25(OH)_2_D increased litter size, prevented fetal resorptions, and increased maternal, fetal, and placental weights [74]. At the higher 1,25(OH)_2_D dose, maternal toxicity characterized by hypercalcemia and weight loss was observed, and fetal and placental weights were reduced compared with solvent-treated controls [74]. This distinction between biochemical correction and physiological response may be particularly relevant in the presence of metabolic dysfunction.

In a non-pregnancy HFD mouse model, VD supplementation attenuated adipose inflammatory signaling, including NF-κB activation, inflammasome-related gene expression, pro-inflammatory cytokine expression, and macrophage recruitment [57]. VD can therefore retain anti-inflammatory activity under HFD-induced metabolic stress; however, whether supplementation restores broader disturbances in VD distribution, metabolism, or tissue responsiveness remains unclear. Improvement in circulating VD status therefore does not necessarily indicate restoration of tissue-level VD regulation, underscoring that circulating 25(OH)D concentrations and tissue-level VD activity represent related but distinct aspects of VD biology [40,51,52,53]. This distinction highlights the importance of examining VD regulation within individual tissues, where VDR and VD-metabolizing enzymes contribute to local signaling capacity.

## 6. Tissue-Specific Developmental Programming

The developmental consequences of maternal metabolic stress are unlikely to occur uniformly across organ systems because tissues differ in their metabolic functions, developmental trajectories, and capacity for local VD signaling. VDR and VD-metabolizing enzymes are distributed across multiple tissues, permitting local regulation of VD activity according to tissue-specific physiological demands [36,75]. Maternal WD exposure can also influence metabolic, inflammatory, and regulatory pathways across developing tissues. Although direct evidence linking maternal WD exposure to altered offspring VD signaling within individual tissues remains limited, evidence from maternal WD/HFD and VD studies identifies several organ systems in which these pathways may intersect. Table 1 summarizes the developmental studies supporting these tissue-specific relationships and distinguishes between evidence directly assessing maternal dietary exposure and offspring VD-related outcomes within the same model and evidence derived from complementary maternal-diet and developmental VD studies.

### 6.1. Colon and Intestinal Immune Development

The intestine represents one of the clearest sites at which maternal dietary exposure and VD signaling may converge. VD contributes to intestinal epithelial barrier integrity, antimicrobial defense, and immune regulation through VDR-mediated transcription [86,87]. Prior work from our laboratory using a Sprague Dawley rat model demonstrated that maternal high-fat, high-sucrose (HFS) diet exposure during pregnancy and lactation, in the absence of maternal obesity, influenced offspring gut microbial composition, inflammatory regulation, and colonic VD-responsive gene expression [76]. Teoh et al. reported altered gut microbiota and higher circulating IL-1β concentrations in offspring exposed to maternal HFS compared with offspring born to control-fed dams [76]. At weaning, maternal HFS-exposed offspring also exhibited increased colonic *Vdr* expression and increased expression of the VDR target cathelicidin; however, neither differed significantly between maternal diet groups in adulthood [76].

Maternal HFD models provide mechanistic context for the relationship between gut microbial composition and systemic inflammation. Cani et al. demonstrated that HFD-induced changes in gut microbiota were associated with increased intestinal permeability and circulating lipopolysaccharide (LPS) [88,89]. Chronic low-dose LPS exposure also reproduced several features of the HFD-associated inflammatory and metabolic phenotype [88]. Within the maternal environment, Gohir et al. similarly demonstrated that HFD altered the maternal gut microbiota, reduced maternal intestinal barrier integrity, and increased circulating maternal LPS and TNF [27]. HFD-exposed fetuses also exhibited differences in intestinal immune and stress-response signaling, including increased NF-κB activation and *Tlr4* expression, although reduced fetal barrier function was not directly demonstrated [27]. Maternal VD exposure can influence some of the same intestinal pathways. In an obesogenic mouse model, higher maternal VD intake was associated with lower intestinal permeability and circulating LPS in male offspring, providing evidence that maternal VD availability can modify offspring gut-barrier integrity and systemic LPS exposure [77]. Thus, evidence from maternal HFS/HFD and VD models converges on intestinal pathways involving microbial composition, epithelial barrier function, inflammatory signaling, and local VDR-responsive processes. In contrast to this complementary evidence, the differences in offspring colonic VD-responsive gene expression observed following maternal HFS exposure provide a more direct connection between maternal dietary exposure and developmental VD signaling within the intestine [76].

### 6.2. Liver

The liver occupies a distinct position in this framework because it is both a central metabolic organ and the primary site of CYP2R1-mediated 25-hydroxylation of VD. Maternal HFD exposure can alter hepatic development independently of overt maternal obesity or diabetes. In a non-human primate model, chronic maternal HFD exposure produced approximately threefold greater fetal hepatic triglyceride accumulation, increased hepatic oxidative stress, and elevated expression of gluconeogenic enzymes and transcription factors. Hepatic triglyceride concentrations remained elevated at 6 months of age, indicating persistence beyond fetal life [78]. Notably, switching HFD-fed mothers to a low-fat diet before a subsequent pregnancy reduced fetal hepatic triglyceride accumulation and partially normalized gluconeogenic gene expression without changing maternal body weight, further separating the effects of maternal diet from maternal adiposity [78].

Beyond its effects on hepatic lipid accumulation and glucose regulation, maternal WD exposure can influence inflammatory and regulatory pathways within the developing liver. In baboons exposed to maternal WD, fetal liver miRNA profiles were altered in parallel with maternal microbiome changes, including miRNAs involved in immune and metabolic regulation [35]. In a separate mouse model of WD-induced maternal obesity, offspring exhibited reduced circulating microbial-derived indole metabolites and lower hepatic *Il10* expression at weaning. Fecal microbial transfer from WD-fed pregnant dams to chow-fed germ-free lactating dams after parturition reproduced this pattern in the offspring, supporting a role for maternal microbial transmission in altered hepatic AHR-related immune signaling [90]. Maternal VDD can affect overlapping hepatic metabolic and inflammatory pathways. In male rat offspring exposed to maternal VDD during pregnancy, Zhang et al. reported impaired glucose tolerance and insulin sensitivity at 16 weeks of age [79]. Hepatic IL-1β, IL-6, IL-8, and TNF-α concentrations were elevated from birth through 16 weeks, while hepatic *Iκbα* expression remained reduced across the same period. Methylation at the +331 CpG site of *Iκbα* was also increased at birth and 16 weeks, providing evidence that prenatal VDD can produce persistent changes in inflammatory regulation within the offspring liver [79]. Prior work from our laboratory provides an additional connection between maternal HFS exposure and offspring hepatic VD metabolism. Maternal HFS exposure during pregnancy and lactation was associated with lower circulating 25(OH)D and higher circulating 1,25(OH)_2_D in offspring, together with reduced hepatic *Cyp2r1* expression despite the absence of classical maternal or offspring VDD [76]. Because CYP2R1 catalyzes hepatic 25-hydroxylation, reduced *Cyp2r1* expression provides a direct link between maternal dietary exposure and altered offspring VD metabolism within the liver. Maternal HFD/WD exposure and maternal VDD therefore affect overlapping hepatic processes involving lipid accumulation, glucose regulation, inflammatory signaling, and epigenetic regulation, although the available studies do not establish that HFD-induced hepatic dysfunction is mediated by impaired VD signaling. The liver nevertheless represents a particularly relevant site of potential convergence because maternal dietary exposure can alter both hepatic metabolic function and, in the maternal HFS model, a core component of offspring VD metabolism.

### 6.3. Pancreas

Pancreatic β-cells express VDR, and 1,25(OH)_2_D_3_ can enhance glucose-stimulated insulin secretion through effects on calcium influx and VDR-mediated transcription within islets [41,91,92]. VDR is strongly expressed in pancreatic β-cells, while CYP27B1 expression within pancreatic islets permits local conversion of 25(OH)D to 1,25(OH)_2_D [41,91]. Maternal HFD exposure can program pancreatic structure and function in a sex-dependent manner. In C57BL/6J mice, maternal HFD exposure during gestation and lactation produced glucose intolerance and insulin resistance in adult offspring, with additional metabolic abnormalities in offspring maintained on HFD after weaning [80]. Male offspring exposed to maternal HFD exhibited lower glucose-stimulated insulin secretion, islet area, insulin content, and expression of *Pdx1* (pancreatic and duodenal homeobox 1), a transcription factor important for β-cell development and insulin production, whereas these measures were increased in female offspring [80]. Maternal HFD also increased oxidative stress within pancreatic islets in males but not females, providing a potential explanation for the sex-dependent β-cell response [80].

Maternal VDD can independently alter offspring pancreatic development and insulin secretion. In Wistar Hannover rats, maternal VDD beginning before conception and continuing through gestation and lactation produced sex-dependent alterations in pancreatic β-cell development [81]. By adulthood, circulating 25(OH)D had normalized after offspring consumed a standard diet, yet pancreatic islets isolated from VDD-exposed males exhibited reduced glucose-stimulated insulin secretion, whereas female offspring were protected from this functional impairment [81]. The HFD and VDD models therefore identify pancreatic β-cell structure and insulin secretory function as shared developmental outcomes, while also revealing substantial sex dependence. However, the available studies do not establish that maternal WD-induced pancreatic dysfunction occurs through disruption of local VD signaling. The pancreas remains a plausible site of interaction because maternal HFD can program β-cell dysfunction, maternal VDD can directly impair pancreatic islet development and insulin secretion, and VDR/CYP27B1 are expressed within pancreatic islets. Direct studies measuring offspring pancreatic VD signaling after maternal WD exposure are still needed.

### 6.4. Skeletal Muscle

Skeletal muscle is a major site of insulin-stimulated glucose disposal and therefore plays a central role in whole-body glucose homeostasis. Maternal HFD and WD exposure can program offspring skeletal muscle dysfunction before overt metabolic disease becomes apparent. In a non-human primate model of maternal obesity associated with chronic WD consumption, fetal and juvenile offspring exhibited impaired insulin-stimulated skeletal muscle glucose transport compared with control offspring, with deficits evident before the development of overt obesity or systemic insulin resistance [82]. Maternal HFD exposure can also alter offspring muscle mitochondrial function in a sex-dependent manner. In mice, maternal HFD reduced skeletal muscle oxidative phosphorylation capacity in female offspring, while no comparable reduction was observed in males [93]. Postweaning HFD further decreased oxidative phosphorylation in females but increased oxidative phosphorylation and electron-transfer-system capacity in males, demonstrating divergent mitochondrial responses by sex and postnatal diet [93].

Evidence connecting developmental VD exposure with skeletal muscle outcomes is more limited but includes human data. In the Southampton Women’s Survey, maternal 25(OH)D concentrations at 34 weeks of gestation were positively associated with offspring hand-grip strength at 4 years of age after adjustment for maternal and childhood confounders. Maternal 25(OH)D was also positively associated with percent lean mass in unadjusted analyses, although this relationship did not remain significant after adjustment [83]. Experimental evidence outside maternal developmental models also demonstrates interactions between dietary fat composition and VD status within skeletal muscle. In male rats fed HFD containing equivalent proportions of energy from fat, Trovato et al. reported that a butter-based diet combined with VD restriction produced muscle-fiber hypotrophy, increased expression of the inflammatory cytokine IL-1β, and markedly reduced IGF-1 expression [94]. Within the butter-based HFD groups, VD supplementation was associated with preservation of muscle-fiber morphology and significantly greater IGF-1 expression than VD restriction, although IL-1β expression remained elevated [94]. Maternal dietary programming and VD-related evidence implicate several overlapping aspects of skeletal muscle biology, including insulin responsiveness, mitochondrial function, muscle development, and inflammatory regulation. However, whether maternal WD exposure directly disrupts local VD regulation within offspring skeletal muscle remains unresolved.

### 6.5. Adipose Tissue

Adipose tissue is both an energy-storage depot and an endocrine and immune organ with the capacity for local VD signaling. Human preadipocytes express VDR and CYP27B1, permitting local VD activation and responsiveness. VD signaling also influences adipocyte differentiation and inflammatory regulation [58,59]. Experimental evidence indicates that maternal VDD can influence offspring adipose development. Wen et al. reported greater adipose tissue volume in adult offspring exposed to maternal VDD during gestation, together with increased proliferation and lipid accumulation in preadipocytes isolated from offspring adipose tissue [66]. Genome-wide methylation and RNA-sequencing analyses further identified differences in DNA methylation and gene expression associated with adipogenesis and metabolic regulation [66]. Notably, offspring received a VD-replete diet beginning at birth, yet differences in body weight emerged later in postnatal development and adipose tissue volume was greater in adulthood, demonstrating that the later phenotype remained evident after the period of maternal VDD exposure [66]. Maternal VDD can also modify the inflammatory response of offspring adipose tissue to a postnatal obesogenic environment. Haroun et al. found that maternal VDD combined with postweaning HFD increased inflammatory RNA and miRNA expression, with greater NF-κB-related signaling in white adipose tissue of adult male offspring. Comparable inflammatory pathway activation was not observed in females, demonstrating a sex-dependent response to maternal VDD and postnatal HFD exposure [95].

Maternal HFD exposure independently programs offspring adipose development. Yokomizo et al. reported increased adipose tissue mass and adipocyte hypertrophy in offspring exposed to maternal HFD, together with greater macrophage accumulation in white adipose tissue [80]. These effects were further amplified by postweaning HFD, with more pronounced increases in adipose inflammatory markers in male than female offspring [80]. Adipose tissue is also a major site of VD storage, linking its expansion with systemic VD distribution, while local VDR-dependent signaling provides a separate pathway through which VD can influence metabolic and inflammatory regulation within the tissue [52,58]. Maternal dietary exposure and disrupted VD availability therefore intersect across adipose accumulation, cellular development, and inflammatory regulation, making this tissue an important target for investigating local VD regulation in developmental programming.

### 6.6. Kidney

The kidney differs from the other tissues considered here because it is the principal endocrine site of CYP27B1-mediated conversion of 25(OH)D to 1,25(OH)_2_D and therefore plays a central role in systemic VD homeostasis. VD signaling also directly regulates the renin-angiotensin system; 1,25(OH)_2_D suppresses renal renin transcription through a VDR-dependent mechanism, linking VD signaling with blood-pressure and electrolyte regulation [96]. Maternal HFD exposure can program offspring renal structure and function. In Sprague Dawley rats exposed to a lard-rich maternal diet during pregnancy and suckling, adult offspring exhibited reduced renal renin and Na^+^/K^+^-ATPase activity despite no differences in kidney weight, glomerular number, or glomerular volume [84]. These renal regulatory changes accompanied the hypertensive phenotype previously described in this model, indicating functional programming in the absence of gross renal structural deficits [84]. Maternal HFD exposure has also produced later renal structural and filtration abnormalities in Wistar rat offspring, including increased glomerular tuft and renal corpuscle area and diet-dependent changes in GFR and urinary protein excretion [97].

Maternal VDD directly affects renal developmental and regulatory pathways. In Sprague Dawley rats, maternal VDD increased fetal renal renin expression, and renal renin remained nearly twofold higher in male offspring at 38 weeks despite consumption of a VD-replete diet after weaning [85]. Renal function assessed at 33 weeks showed reduced creatinine clearance and solute excretion and evidence of sodium retention in males, whereas females showed increased water intake and urine output without impaired creatinine clearance [85]. In a separate mouse model, maternal VDD delayed glomerular maturation and extended the period of nephrogenesis in offspring, accompanied by reduced glomerular diameter and increased renal renin and angiotensin II type 1 receptor expression [98]. Offspring also developed higher blood pressure and early indices of altered renal function [98]. Human data provide more cautious support for developmental effects of prenatal VD status on renal function. In the Generation R cohort of 4,212 mother-child pairs, maternal second-trimester 25(OH)D was not consistently related to childhood kidney volume and was not associated with microalbuminuria, but higher maternal 25(OH)D was associated with a modestly lower creatinine-based estimated GFR at approximately 6 years of age. The authors characterized these observations as hypothesis-generating [99]. Maternal HFD and VDD therefore both influence renal pathways involved in filtration, electrolyte handling, and renin-angiotensin regulation, although the direction and magnitude of the responses vary by experimental model. Because the kidney is also the major endocrine site of VD activation, developmental alterations within this organ are particularly relevant to systemic VD homeostasis. However, current evidence does not establish that maternal WD directly alters offspring renal CYP27B1 activity or other components of renal VD signaling. This gap distinguishes renal developmental programming from direct evidence of WD-induced disruption of the renal VD pathway.

#### Emerging Patterns Across Tissues

Several patterns emerge across these organ systems. First, maternal HFD/HFS exposure can produce developmental alterations that persist beyond the period of maternal dietary exposure. In some models, differences remain evident despite consumption of a control diet after weaning, including changes in offspring VD status and pancreatic metabolic function [76,80]. Maternal dietary exposure has also been associated with developmental alterations across the liver, skeletal muscle, intestine, adipose tissue, and kidney, although the timing, persistence, magnitude, and affected pathways differ among tissues [27,78,82,93,97]. Second, recurring biological processes include inflammatory dysregulation, oxidative stress, altered nutrient and glucose metabolism, and mitochondrial dysfunction, but no single mechanism is expressed uniformly across organs. Maternal HFD/WD exposure has been associated with oxidative and metabolic dysfunction in fetal liver, sex-dependent oxidative stress in pancreatic islets, impaired insulin-stimulated glucose transport and mitochondrial respiration in skeletal muscle, altered intestinal immune and barrier-related signaling, and renal regulatory dysfunction [27,78,80,82,84,93,97]. Third, VD-responsive pathways intersect with many of the same metabolic and inflammatory processes, but the strength of evidence for direct disruption of local VD regulation by maternal diet differs substantially among tissues. The clearest developmental evidence is available in tissues in which maternal dietary exposure has been linked directly with components of the VD pathway, including altered colonic VD-responsive signaling and hepatic *Cyp2r1* expression following maternal HFS exposure [76]. In other tissues, maternal dietary programming and VD-related regulation have largely been demonstrated in separate experimental models. Maternal HFD exposure has been shown to program pancreatic, skeletal muscle, adipose, and renal outcomes [80,82,84,97]. Independent studies demonstrate VD-sensitive or VDR-dependent regulation within these organ systems, although the nature and strength of the evidence differ among tissues [58,59,80,82,85,96]. This overlap identifies a mechanistic question requiring direct assessment of VD-related regulation within individual tissues. Because effective VD signaling depends on metabolite availability, enzymatic activation and degradation, and VDR-mediated transcription, tissue-specific evaluation of VDR and VD-metabolizing enzymes may provide information that cannot be inferred from circulating 25(OH)D concentrations alone [40,51,52,53]. Determining whether maternal dietary exposure produces persistent, tissue-specific changes in offspring VD regulation therefore remains an important unresolved question.

## 7. Bridging Maternal Dietary Patterns to Offspring Vitamin D Status

Despite growing evidence linking maternal dietary exposure, metabolic stress, and VD biology, it remains unclear whether maternal WD exposure disrupts VD signaling in offspring independently of classical VDD. Much of the developmental literature has examined maternal VDD or supplementation and their relationships with circulating VD status and offspring outcomes [2,72]. In contrast, evidence from obesity and HFD models indicates that metabolic dysfunction can affect VD distribution and storage, hepatic 25-hydroxylation, and components of local VD regulation even when inadequate VD intake does not fully account for reduced circulating 25(OH)D [51,52,53]. Circulating 25(OH)D concentrations may therefore provide an incomplete representation of VD regulation under conditions of metabolic stress. VDR and VD-metabolizing enzymes exhibit tissue-specific expression [36,41]. Maternal HFD/WD exposure can likewise program metabolic and inflammatory pathways across multiple offspring organ systems [26,28,82]. Direct assessment of local VD metabolism and VDR-mediated regulation within offspring tissues following maternal WD exposure remains limited. Consequently, it remains unclear whether maternal WD exposure produces persistent, tissue-specific alterations in offspring VD regulation, and whether such changes occur alongside inflammatory dysregulation in metabolically distinct tissues. Addressing this gap may distinguish changes in systemic VD availability from alterations in local VD metabolism and VDR-mediated regulation within an adverse maternal metabolic environment.

## 8. Conclusions

Maternal dietary composition represents an important component of the developmental environment, with WD exposure associated with metabolic, inflammatory, placental, and regulatory disturbances that may influence long-term offspring health. VD is particularly relevant within this environment because its biological activity depends not only on circulating 25(OH)D availability but also on tissue distribution, enzymatic metabolism, and VDR-mediated signaling. The literature reviewed here identifies substantial overlap between metabolic and inflammatory pathways associated with maternal WD exposure and pathways involved in VD metabolism and signaling across metabolically active tissues. However, direct evidence that maternal WD alters offspring tissue-specific VD pathways remains limited. Examining local VD metabolism and VDR-mediated regulation within individual tissues may therefore identify developmental changes that cannot be inferred from circulating VD status alone and help clarify whether VD signaling contributes to offspring metabolic and immune programming.

## Figures and Tables

**Figure 1:**
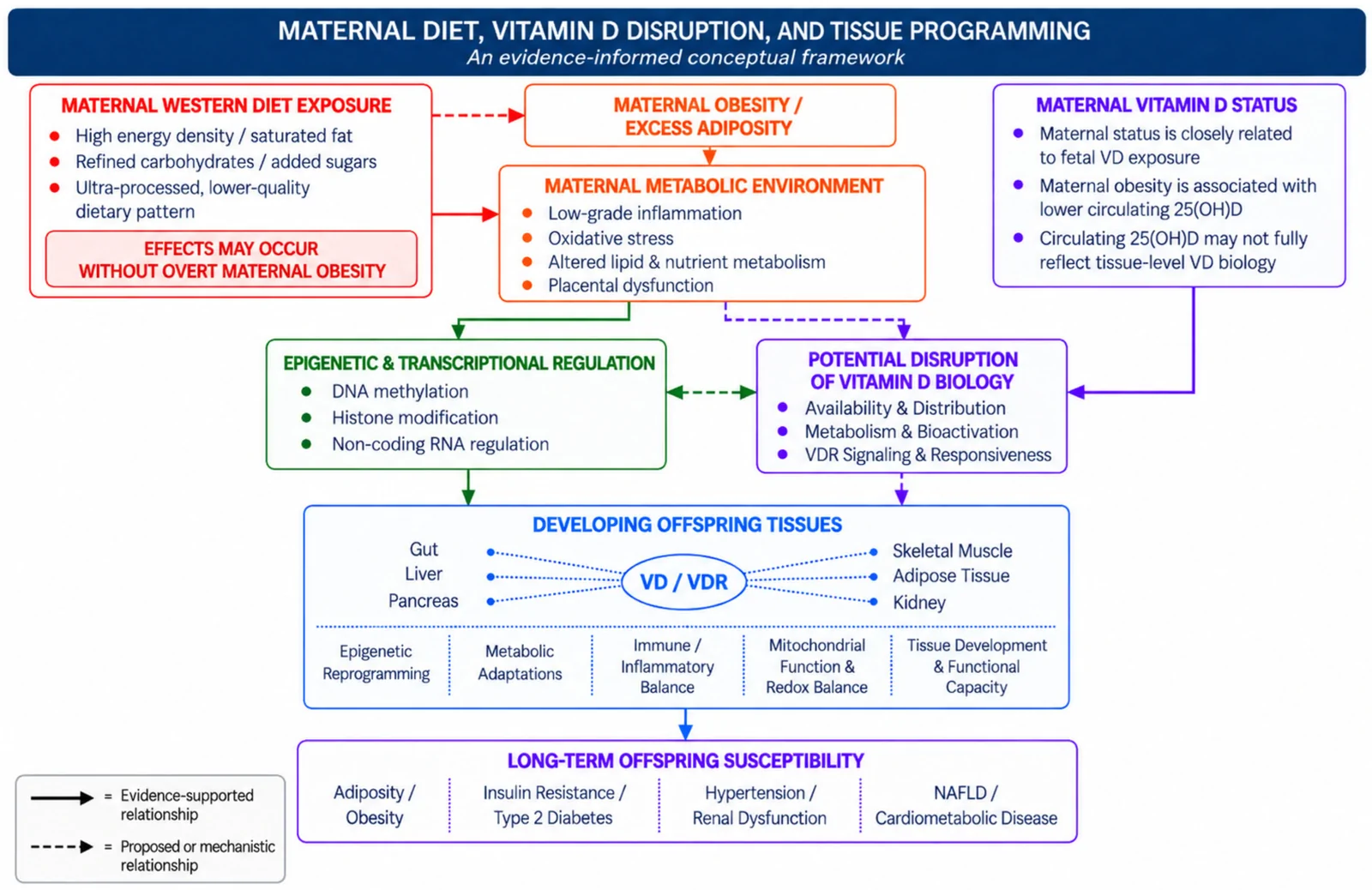
Proposed framework linking maternal Western diet exposure with developmental programming and vitamin D regulation. Maternal Western diet (WD) exposure can contribute to metabolic and inflammatory conditions within the maternal environment and influence developmental programming through placental, metabolic, inflammatory, and epigenetic pathways. In parallel, WD-associated metabolic dysfunction may influence vitamin D (VD) availability and tissue distribution, enzymatic metabolism, and VDR-mediated responsiveness. These pathways may converge within developing offspring tissues; however, direct evidence that maternal WD-induced developmental programming is mediated through disruption of tissue-specific VD signaling remains limited. Solid arrows represent relationships supported by direct or established evidence within the literature reviewed, whereas dashed arrows indicate proposed or mechanistically supported relationships for which direct developmental evidence remains limited.

**Table 1:** Developmental evidence across maternal dietary exposure, vitamin D regulation, and offspring tissues.

Study/Model	Maternal Exposure	Offspring Tissue	VD-Related Measure	Main Offspring Finding	Evidence
Teoh et al. [76], rat	HFS, pregnancy/ lactation	Colon	*Vdr*; cathelicidin	↑ *Vdr* and cathelicidin at weaning; no adult difference	Direct
Gohir et al. [27], mouse	HFD	Fetal intestine	None	↑ NF-κB activation and *Tlr4*	Maternal diet
Villa et al. [77], mouse	VD in obesogenic model	Intestine	Maternal VD exposure	Higher VD intake associated with ↓ permeability and circulating LPS in males	Developmental VD
Teoh et al. [76], rat	HFS, pregnancy/ lactation	Liver	*Cyp2r1*; circulating VD metabolites	↓ *Cyp2r1* and 25(OH)D; ↑ 1,25(OH)_2_D	Direct
McCurdy et al. [78], NHP	HFD	Fetal liver	None	↑ hepatic triglycerides and oxidative stress; metabolic regulation affected	Maternal diet
Zhang et al. [79], rat	VDD during pregnancy	Liver	Maternal VD availability	Persistent inflammatory/epigenetic changes; impaired glucose regulation	Developmental VD
Yokomizo et al. [80], mouse	HFD, gestation/ lactation	Pancreas/islets	None	Impaired β-cell function and insulin secretion; sex-dependent	Maternal diet
Schavinski et al. [81], rat	VDD, preconception through lactation	Pancreas/islets	Maternal VD availability	Impaired insulin secretion persisted in adult males despite later VD normalization	Developmental VD
Campodonico-Burnett et al. [82], NHP	Maternal obesity + WD	Skeletal muscle	None	Impaired insulin-stimulated glucose transport in fetal/juvenile offspring	Maternal diet
Harvey et al. [83], human cohort	Maternal 25(OH)D	Muscle phenotype	Maternal 25(OH)D	Higher maternal 25(OH)D associated with greater grip strength at age 4	Developmental VD
Yokomizo et al. [80], mouse	HFD	Adipose	None	↑ adiposity, adipocyte hypertrophy, and macrophage accumulation	Maternal diet
Wen et al. [66], rat	VDD, preconception/gestation	Adipose	Maternal VD availability	↑ adipose volume and adipogenesis; epigenetic/transcriptional differences	Developmental VD
Armitage et al. [84], rat	Fat-rich diet, pregnancy/suckling	Kidney	None	↓ renal renin and Na^+^/K^+^-ATPase activity in adult offspring	Maternal diet
Boyce et al. [85], rat	Maternal VDD	Kidney	Maternal VD availability; renal renin	↑ renal renin persisted after postweaning VD repletion	Developmental VD

NOTE: Studies are summarized according to maternal exposure, offspring tissue, VD-related measures, and primary offspring findings. The table includes both direct evidences assessing maternal WD/HFD/HFS exposure and offspring VD-related outcomes within the same developmental model and complementary evidence from maternal-diet and developmental VD studies. Evidence classification: Direct, maternal WD/HFD/HFS exposure with assessment of VD-related outcomes in offspring within the same developmental model; Maternal diet, maternal dietary exposure with relevant offspring tissue outcomes but without direct assessment of VD-related outcomes; Developmental VD, maternal VD status or manipulation with offspring outcomes without testing maternal diet-induced VD disruption. Abbreviations: HFD, high-fat diet; HFS, high-fat, high-sucrose diet; LPS, lipopolysaccharide; NHP, non-human primate; VD, vitamin D; VDD, vitamin D deficiency; WD, Western diet; 25(OH)D, 25-hydroxyvitamin D; 1,25(OH)_2_D, 1,25-dihydroxyvitamin D; ↑ increased; ↓ decreased.

## Data Availability

No new data were created or analyzed in this study. Data sharing is not applicable to this article.
